# Subtle Radiographic Findings of Thoracolumbar Fractures in Patients with Ankylosing Spinal Disease

**DOI:** 10.30476/beat.2021.86642

**Published:** 2021-01

**Authors:** Johannes Gossner

**Affiliations:** 1 Department of Diagnostic and Interventional Radiology, Evangelisches Krankenhaus Göttingen- Weende, An der Lutter 24, 37074 Göttingen, Germany

Ankylosing spinal disease (ASD) is a common clinical entity [1]. The main etiologies of ASD are diffuse idiopathic skeletal hyperostosis (DISH), ankylosing spondylitis and postoperative spinal fusion after removal of instrumentation. Because of the rigidity of the spine patients with ASD are prone to fractures even with minor trauma and unstable fracture patterns (distraction fractures and involvement of the posterior column) are common [1]. Because a substantial number of fractures may be occult on radiography, cross sectional imaging (especially with computed tomography) has been advocated in the diagnosis of these fractures [1-4]. Nonetheless for a variety of reasons emergency physicians, trauma surgeons and radiologists are often faced with trauma radiographs of the thoracic or lumbar spine of patients with ASD. On the one hand radiography is widely available, cheap and fast to perform in trauma settings. On the other hand, especially the existence DISH is often without clinical symptoms and therefore the existence of thoracolumbar ASD is unknown to patients undergoing radiography after trauma. Interpretation of thoracolumbar radiographs in ASD is challenging and the presented clinical images like to highlight the often subtle radiographic findings with CT correlation. If there is any doubt on conventional radiography further work-up with cross sectional imaging (CT/MRI) is necessary (Figures 1-4).

**Fig. 1:**
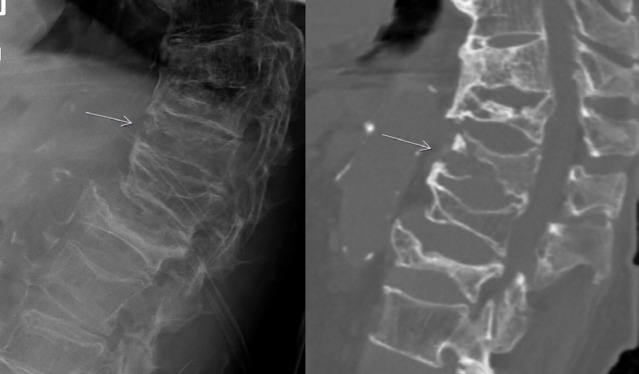
Distraction fracture in an 88-year-old women. She presented with a femoral fracture and spinal pain was reported in the clinical course. Radiography (on the left) shows signs of DISH, old compression fractures and an interruption of the ventral cortex of TH 11. The correlating CT (on the left) shows the complex distraction injury of the thoracolumbar junction and the old compression fractures

**Fig. 2:**
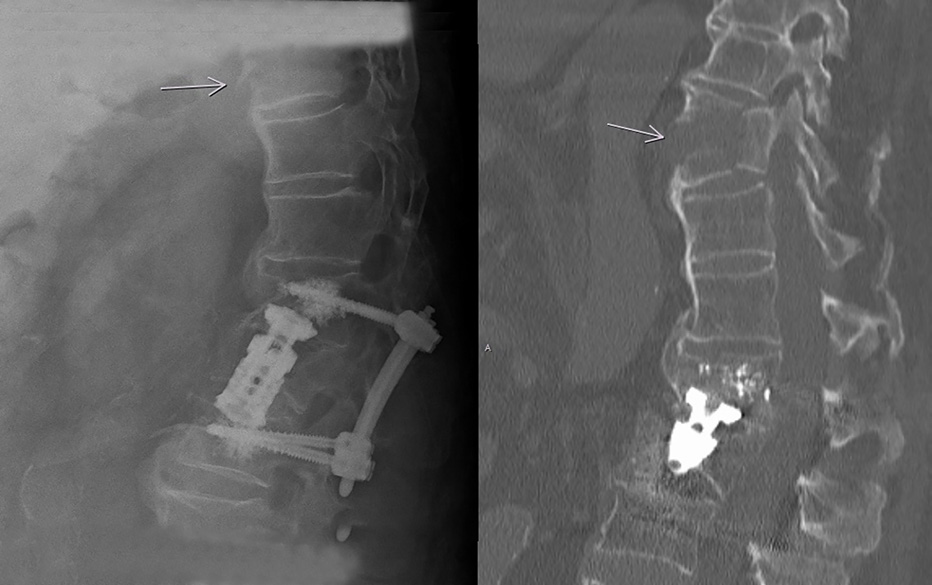
Fracture in an 87-year-old man presenting with immobilizing lumbar pain after a fall. Radiography (on the left) shows spondylodesis of a L2- fracture. At the border of the x-ray a step in the ventral cortex of TH 10 and a subtle fracture line of the vertebral body can be found. The performed CT-scan (on the right) shows the unstable distraction fracture

**Fig. 3:**
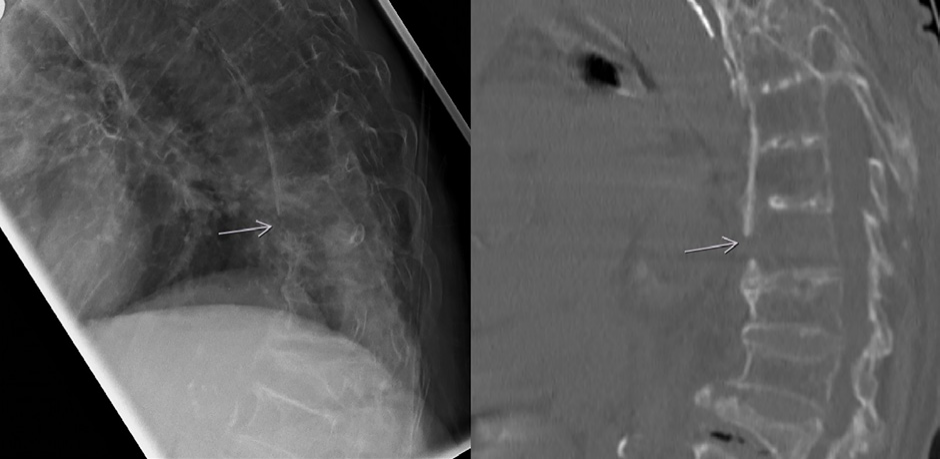
Fracture in an 88-year-old women presenting with persitent pain after a fall on the ground. Radiography (on the left) shows a disruption in the calcified ventral longitudinal ligament. This finding is confirmed on the CT-scan (on the right)

**Fig. 4:**
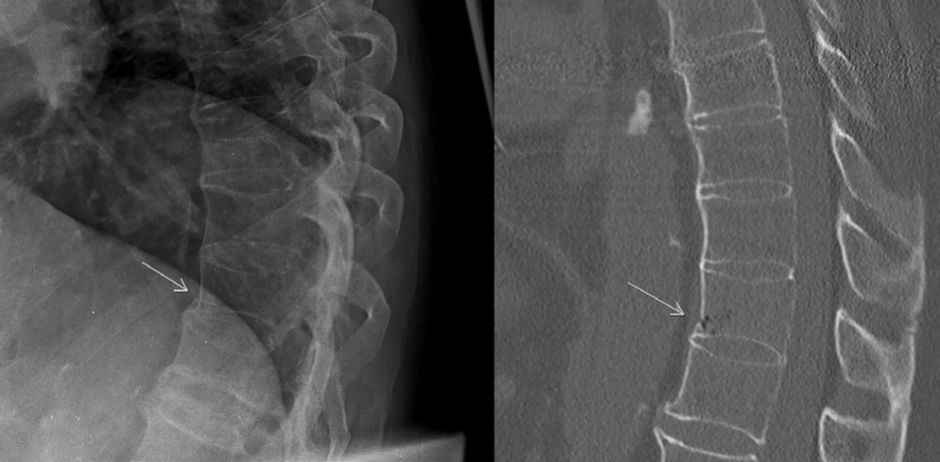
Fracture in a 70-year-old men with known ankylosing spondylitis. Radiography (on the left) shows only a very subtle kinking at the ventral cortex of TH 11. The CT-scan (on the right) confirms this finding

## Conflict of Interest:

None declared.
